# Metabolome-Wide Analysis of Stable Isotope Labeling—Is It Worth the Effort?

**DOI:** 10.3389/fphys.2015.00344

**Published:** 2015-11-20

**Authors:** Daniel Weindl, Andre Wegner, Karsten Hiller

**Affiliations:** Luxembourg Centre for Systems Biomedicine, University of LuxembourgEsch-sur-Alzette, Luxembourg

**Keywords:** stable isotope labeling, non-targeted metabolomics, Metabolic Flux Analysis (MFA), metabolism, 13C

Stable isotope assisted metabolomics techniques have emerged as a valuable tool in systems biology for metabolic flux analysis and pathway discovery (Duckwall et al., 2013; Chokkathukalam et al., 2014; Niedenführ et al., 2015). Traditionally, stable isotope labeling analyses have been highly targeted, meaning that isotopic enrichment of only a small set of metabolites has been analyzed to deduce metabolic fluxes. However, in recent years, tools for the global non-targeted detection, quantification, and computational analysis of isotopic enrichment have become available (Hiller et al., 2010, 2013; Bueschl et al., 2012; Creek et al., 2012; Chokkathukalam et al., 2013; Cho et al., 2014; Huang et al., 2014). In this article, we will discuss whether and how such novel non-targeted stable isotope labeling analyses can be applied for systems-biomedical research.

Pathological alterations of cellular processes usually manifest in altered metabolism. Therefore, analysis of metabolism is an ideal entry-point to diagnose or analyze diseases. Metabolic fluxes are the endpoint of cellular regulation and most likely to reflect changes on the genome, transcriptome or proteome level, and hence, are a valuable read-out for biomedical research (Wegner et al., 2015). Since intracellular metabolic fluxes cannot be measured directly, they are probed using stable isotope labeling: An isotopically enriched substrate is applied and the metabolization of this tracer leads to isotopic enrichment in downstream metabolites, depending on the underlying metabolic fluxes (Buescher et al., 2015). These enrichment patterns are analyzed by mass spectrometry (MS) or nuclear magnetic resonance (NMR) and are used to deduce metabolic fluxes (Truong et al., 2014; Young et al., 2014).

Because isotopic labeling patterns are a direct consequence of metabolic fluxes, changes in these patterns indicate metabolic flux changes (Sauer, 2006). Consequently, global analysis of labeling patterns would allow for the global detection of metabolic flux changes. Because isotopic enrichment can be deduced solely from mass spectra (Jennings and Matthews, 2005), differential flux analysis based on differences in these isotopic labeling patterns does not require prior compound identification or a model of the metabolic network. This way, a non-targeted stable isotope labeling analysis can also consider unexpected or unknown compounds and reactions, circumventing current limitations of compound identification. Such a data-driven metabolic flux analysis is perfectly suited to pinpoint disease-specific alterations of cellular metabolism as little information on the experimental outcome is required. However, even in cases where there are specific hypotheses in place, a data-driven analysis may identify previously overlooked features. As such, non-targeted stable isotope labeling analysis can function as a hypothesis generator, aiding the design of subsequent experiments. Other than e.g., ^13^C metabolic flux analysis (^13^C-MFA) or flux balance analysis (FBA) which completely rely on the integrity of the underlying metabolic network and its constraints, such a non-targeted analysis does not require much biochemical *a priori* information and is, therefore, not biased by the limited knowledge of the metabolic network.

The experimental effort for a non-targeted stable isotope labeling study is not much higher than for a conventional targeted analysis; the main difference is that a non-enriched sample is required as reference to determine isotopic enrichment in the sample of interest (Figure 1A; Weindl et al., 2015). In terms of analytical requirements, the non-targeted analysis is similar to label-free non-targeted metabolomics: The wide range of concentrations and chemical heterogeneity of metabolites pose problems to the metabolome-wide analysis of isotopic enrichment. Therefore, sensitive and robust analytics and powerful ion-chromatographic deconvolution and spectrum matching algorithms are required to accurately quantify also low-intensity isotopic peaks (Wegner et al., 2013). Yet, isotopic labeling patterns are much more robust towards technical variation during sample workup than metabolite levels, because the labeled and unlabeled species are subject to the same biases, thus increasing comparability of different measurements. In comparison to targeted stable isotope labeling analyses, one faces the problem of lower sensitivity because single ion (SIM) or selected reaction monitoring (SRM) cannot be used, as the analytes of interest are not known beforehand. Apart from that, a non-targeted approach would yield information on the same analytes as a targeted analysis, but also include additional compounds.

**Figure 1:**
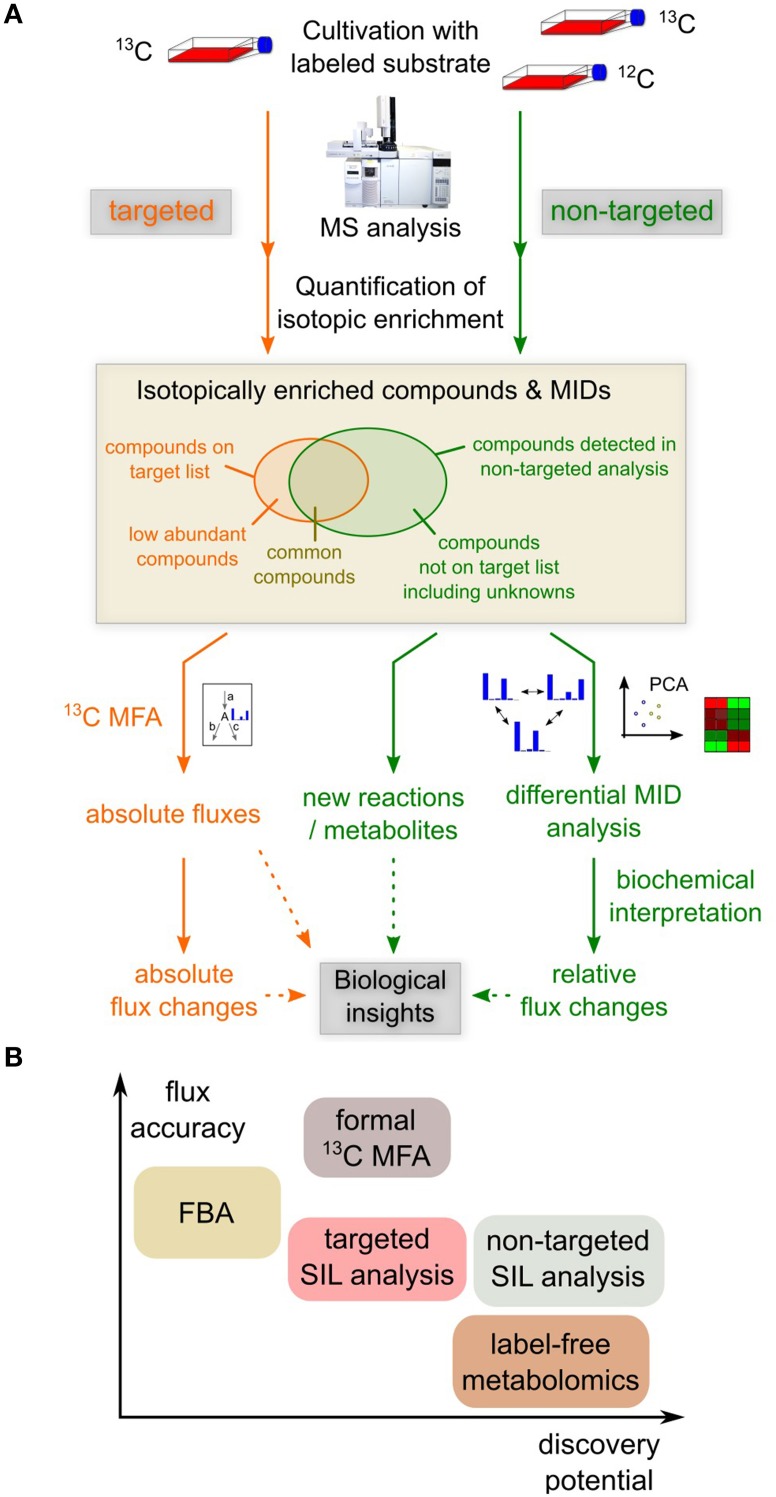
**Different approaches for metabolic analyses**. **(A)** Targeted and non-targeted analysis of stable isotope labeling (SIL). Non-targeted detection of isotopic enrichment requires the measurement of an enriched and a non-enriched sample. Targeted approaches usually have higher sensitivity toward target analyses, but non-targeted analyses generally yield information on more compounds. Targeted ^13^C-MFA provides absolute flux estimates for a given metabolic network model, but does not provide information on unknown reactions or metabolites. Non-targeted stable isotope labeling analysis can point to novel metabolic reactions and metabolites. Differential mass isotopomer distribution (MID) analysis reveals changes in metabolic fluxes in a data-driven manner. However, the mechanistic interpretation is more complex and requires in-depth biochemical knowledge. **(B)** Comparison of different metabolic analyses in terms of flux accuracy they provide and to which extent they allow for the discovery of novel reactions or metabolites. Non-targeted methods have a higher discovery potential, but fail to provide accurate absolute flux information.

If non-targeted stable isotope labeling analysis is that powerful, why is not more widely applied? There is a great potential and the experimental effort is manageable, however, the subsequent data interpretation is much more complex. Changes in isotopic enrichment can be detected easily and do not require any biochemical knowledge (Huang et al., 2014). However, interpreting these changes in terms of underlying metabolic flux changes requires a deep understanding of cellular metabolism and is further complicated by the usually high number of compounds, many of which remain unidentified. Furthermore, although algorithms for the non-targeted and quantitative detection of isotopic enrichment in MS data have been around for a couple of years, non-targeted analysis of stable isotope labeling is still hampered by the lack of dedicated and intuitive software tools. For these reasons, only a few studies have successfully applied non-targeted stable isotope labeling analysis to provide novel biological insights, underlining the higher complexity of data interpretation and the need for more advanced tools and algorithms.

*In vivo* application of non-targeted stable isotope labeling analysis is technically possible, but data analysis is complicated by the metabolic complexity of a whole organism. For that reason, the main field of application would be *in vitro* setups with e.g., cell lines or patient-derived primary cells which are often applied in biomedical research. In such a context, non-targeted stable isotope labeling has great potential to reveal hitherto overlooked metabolic pathways or metabolic flux changes. Although such non-targeted approaches are not meant to replace targeted methods and cannot provide absolute flux information (Figure 1B), they provide means to get closer to the full picture.

In summary, if the aforementioned limitations can be dealt with, non-targeted stable isotope labeling analysis is worth the effort and a valuable addition to the systems biology toolbox. This rather unbiased approach can help to detect flux changes in regions of the metabolic network which were not expected to be affected or are not known yet, thereby deepening the understanding of pathobiochemical mechanisms. This understanding is the basis for the development of novel diagnostic and therapeutic methods that will impact human health. Further improvements in sensitivity and specificity of current algorithms and the development of easy-to-use software tools will help to realize the great potential of non-targeted stable isotope labeling analysis.

## Author contributions

AW and DW jointly wrote the manuscript. KH critically revised the manuscript.

## Funding

DW and KH are supported by the Fonds National de la Recherche (FNR) Luxembourg (ATTRACT A10/03).

### Conflict of interest statement

The authors declare that the research was conducted in the absence of any commercial or financial relationships that could be construed as a potential conflict of interest.
